# Syringing the Ear

**Published:** 1901-08-17

**Authors:** 


### SYRINGING THE EAR.

Dr. Langstaff[1] describes a contrivance which he has made for syringing the ear. He is bold enough to call it a "new" device, but whether new or not it seems sufficiently useful to deserve notice. It may be attached to any bag or fountain syringe, and consists of an ordinary tubular metal ear-speculum of medium size, along the upper wall of which (within the speculum) runs a small tube which terminates at its inner end in a small slit-like opening and at its outer end in a bulbous expansion for the attachment of a rubber tube connecting it with the syringe. On this tube a stopcock is arranged which until it is pressed upon prevents the water flowing. If then the speculum be introduced in such a position that the side which carries the inner tube is uppermost, on pressing the stopcock the water will flow towards the tympanum just where it is wanted—namely, along the upper wall of the meatus. Of course this arrangement is of no use for the removal of foreign bodies and suchlike proceedings, but for the regular cleansing of the ear by the patient or the nurse it is likely to prove very effective, having all the safety which is sought to be ensured by the various stopping devices with which ear syringes are often provided, besides the great advantage of directing the flow of the water in the best direction for securing the proper washing out of the tympanum. The speculum may also be provided with a drain tube, which may be found useful when the patient is lying in bed, but will probably be found only a complication under other circumstances.

[1] New York Med. Record, July 6.

